# Beyond Detection: Comparing State-Based Newborn Screening Methods for Effective Mucopolysaccharidosis I Diagnosis

**DOI:** 10.3390/ijns12010015

**Published:** 2026-03-03

**Authors:** Rithika Thampy, Nishitha R. Pillai, Michael Evans, Chester B. Whitley, Paul J. Orchard, Matthew Ellinwood, Amy Gaviglio

**Affiliations:** 1 Robbins College of Health and Human Sciences, Baylor University, Waco, TX 76706, USA; 2 Advanced Therapies Program, Division of Genetics and Metabolism, Department of Pediatrics, University of Minnesota, Minneapolis, MN 55454, USA; 3 Clinical and Translational Science Institute, University of Minnesota, Minneapolis, MN 55414, USA; 4 Division of Pediatric Blood and Marrow Transplantation, Department of Pediatrics, University of Minnesota, Minneapolis, MN 55454, USA; 5 National MPS Society USA, P.O. Box 14686, Durham, NC 27709-4686, USA; 6 Connetics Consulting, LLC., Minneapolis, MN 55417, USA; 7 Association of Public Health Laboratories, 7700 Wisconsin Avenue, Suite 1000, Bethesda, MD 20814, USA

**Keywords:** MPS I, newborn screening, glycosaminoglycans

## Abstract

Mucopolysaccharidosis type I (MPS I) results in the accumulation of glycosaminoglycans (GAG) and, for the purposes of newborn screening, is differentiated into two forms: severe (Hurler syndrome) versus attenuated (encompassing Scheie and Hurler-Scheie syndromes). MPS I was added to the federal Recommended Uniform Screening Panel for newborn screening (NBS) in 2016, and as of December 2025, 45 of 54 programs in the United States (US) screen for MPS I. Within the newborn screening program, a second-tier analysis of GAG is thought to reduce false-positive rates, particularly through mitigating the detection of pseudodeficiency. However, there have been some concerns that the use of second-tier GAG analysis might inadvertently result in missed detection of attenuated cases. A survey of all US NBS programs was conducted requesting data on the total number of screen-positive NBS results for MPS I as well as the final diagnostic outcome from these results. Diagnostic outcomes after screening were classified as false-positive, pseudodeficiency, severe MPS I, attenuated MPS I, and MPS I of undetermined phenotype. Additionally, information on testing methodologies and dates of MPS I NBS implementation was collected. Responses were obtained from 32 NBS programs. The cohort of screening programs utilizing second-tier blood spot GAG determinations detected a higher proportion of severe cases than those not using this second-tier test (48% vs. 29%). The proportion of attenuated cases remained consistent between both groups (13% vs. 14%). The proportion of pseudodeficiency detection was only slightly lower in the cohort using second-tier GAG analysis (85% vs. 91%). Second-tier GAG analysis appears to reduce the detection of false-positive cases and improves the resolution of severe MPS I cases, though the proportion of pseudodeficiency was only slightly lower compared to the programs that do not use second-tier GAG analysis. Currently, the proportion of attenuated cases is comparable between the two cohorts, but the higher number of “undetermined phenotype” cases may eventually shift the balance toward states not using GAG analysis once the type is determined.

## 1. Background

Mucopolysaccharidosis type I (MPS I) is a rare autosomal recessive disorder caused by a deficiency of the enzyme a-L-iduronidase (IDUA), resulting in systemic accumulation of glycosaminoglycans (GAG), including heparan and dermatan sulfates. Clinically, MPS clinical phenotypes include the severe, neuronopathic type, formerly known as Hurler syndrome (McKusick’s OMIM #607014), as well as a more attenuated form previously referred to as Scheie syndrome (OMIM #607016) and/or Hurler-Scheie syndrome (OMIM #607015). In the context of newborn screening, those with Hurler syndrome require treatment with hematopoietic stem cell transplantation (HSCT). This is distinguished from those with the attenuated form for whom transplant is not required due to the relative lack of neurodevelopmental deterioration. Without early diagnosis and intervention, affected individuals may have a delay in establishing the diagnosis resulting in irreversible damage to multiple organ systems [1,2,3]. Because the efficacy of treatment for MPS I is closely linked to the timing of intervention, early identification through newborn screening (NBS) is critical. In recognition of this, MPS I was added to the Recommended Uniform Screening Panel (RUSP) in 2016, and as of December 2025, 45 of 54 NBS programs in the United States have included it in their screening panels [4].

All NBS programs screening for MPS I in the US use a first-tier assay that measures a-iduronidase enzymatic activity in dried blood spots using tandem mass spectrometry or fluorometric assays [5]. However, these methods lacks sufficient specificity to differentiate pseudodeficiency alleles, which may lead to false-positive screening results and unnecessary clinical referrals [6]. To improve specificity, some states have added a second-tier test that measures glycosaminoglycans (GAG), which are presumed to accumulate only in true MPS I (and other mucopolysaccharidosis) cases [7]. In programs that utilize this multi-tier screening approach, only those screening results with decreased IDUA enzyme activity and elevated GAGs are considered screen-positive. While the use of this second-tier test is intended to reduce false-positive results, there remain concerns that, in attenuated MPS I phenotypes, GAG accumulation may be minimal or delayed, which might increase the risk of missing individuals with the attenuated form of MPS I when GAG analysis is part of the screening algorithm.

This study examines how second-tier internal disaccharide GAG testing might impact detection rates in MPS I newborn screening. Programs with and without GAG testing as part of their screening algorithm were compared using survey data on false-positives, pseudodeficiency, and confirmed MPS I cases.

## 2. Methods

### 2.1. Study Design and Data Collection

This was a cross-sectional study of US NBS programs that screen for MPS I as of August 2024 (*n* = 45). A standardized survey was distributed by the Association of Public Health Laboratories (APHL) listserv to program directors of each state. Programs were asked to report cumulative screen-positive and diagnostic outcome data over the entire time they have been screening for MPS I. These data were then classified into five categories: confirmed severe MPS I, confirmed attenuated MPS I, pseudodeficiency, false-positive, or confirmed MPS I, undetermined phenotype. Survey outcomes were categorized as false-positive or true-positive based on confirmatory clinical evaluation, which typically included alpha-iduronidase enzyme activity testing, urine GAG analysis, and molecular testing of the IDUA gene. True-positive cases were further classified as severe or attenuated MPS I using a combination of molecular testing, clinical examination, imaging findings, and treatment courses. An additional “unknown” category included children with confirmed biallelic IDUA variants who could not be clearly classified into either phenotype, particularly when the genotype was not predictive of clinical severity. In this study, pseudodeficiency cases were included within the true-positive group, as these individuals exhibit reduced enzyme activity in vitro that would appropriately trigger newborn screening detection; this was not considered a technical limitation of the screening process.

In states that incorporate molecular testing methodologies such as Sanger sequencing or next-generation sequencing (NGS) into the newborn screening (NBS) process, categorization into the different groups could be done using NBS results. In states that do not include molecular testing as part of NBS, categorization was completed during confirmatory testing that includes molecular analysis of the IDUA gene. All infants ultimately underwent molecular analysis either through NBS or subsequent confirmatory testing. This molecular testing result plays a critical role in phenotype classification, as clinical manifestations—particularly in non-severe MPS I—may not be apparent during infancy.

Respondents also provided details on their screening protocols, including the year of MPS I NBS implementation, first-tier testing method, and whether they used second-tier GAG testing. For the programs that used second-tier GAG analysis, information was collected on the GAG testing methods. At the time this survey was done, all states that were using second-tier GAGs were using internal disaccharide testing methodology by liquid chromatography–tandem mass spectrometry (LC-MS/MS). Data from the Newsteps.org database were also reviewed to confirm survey responses [4].

### 2.2. Cohort Comparison and Analysis

Programs were divided into two cohorts based on their screening approach: those using second-tier GAG analysis (*n* = 8) and those using first-tier enzyme only testing (*n* = 17). The distribution of outcomes (pseudodeficiency, confirmed MPS I (severe or attenuated), false-positives, and confirmed MPS I, unknown phenotype) were compared between the two groups using Fisher’s exact test. Positive predictive value (PPV) was then calculated as the proportion of confirmed MPS I diagnoses that included severe, attenuated, and undetermined phenotype cases out of all the screen-positive results.

## 3. Results

A total of 32 US NBS programs provided responses regarding their methodology/outcomes related to MPS I screening (Table 1). Programs were divided into two cohorts based on whether they implemented second-tier internal disaccharide GAG analysis or not.

**Figure 1:**
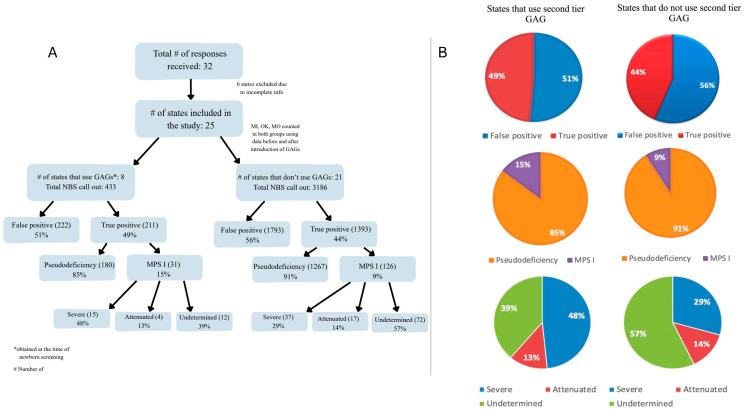
Comparative outcomes in MPS I screening programs with and without second-tier GAG testing. (**A**) Breakdown of cohort comparison methodology between programs using enzyme-only testing and those using second-tier GAG analysis. (**B**) Pie charts display the distribution of outcomes in each cohort.

Among all MPS I cases, the states that used second-tier GAG testing appeared to have a higher proportion of confirmed severe MPS I cases, i.e., Hurler syndrome, compared to states that relied solely on first-tier enzyme analysis, though this was not statistically significant. (48% vs. 29%, *p* = 0.06; Figure 1). In contrast, the proportion of attenuated cases was nearly equivalent between the two cohorts (13% vs. 14%, *p* = 1.00; Figure 1). Notably, the group that used GAG analysis showed a lower, though not statistically significant, proportion of cases with an unknown phenotype (39% vs. 57%, *p* = 0.07; Figure 1).

The proportion of pseudodeficiency cases among true-positive cases was lower in states using GAG analysis (85% vs. 91%, *p* = 0.01).

## 4. Discussion

This multi-state analysis of NBS outcomes for MPS I provides valuable insight into the utility and potential limitations of using second-tier GAG testing within state-based NBS algorithms. As more programs adopt tiered testing strategies to improve screening accuracy and reduce unnecessary follow-up, understanding the impacts of these approaches on attenuated or milder disease forms becomes critical.

The addition of second-tier GAG testing was associated with a higher proportion of confirmed severe MPS I cases (48%), compared to those using enzyme activity alone (29%). Even though this did not reach statistical significance when compared across the MPS I phenotypic spectrum, the states that used second-tier GAGs had a significantly higher yield of severe cases per call-out (3.5%) when compared to the states that did not use second-tier biochemical screening (1.1%), with a *p* value of <0.001. This likely reflects the greater specificity of GAG analysis in detecting true disease with early substrate accumulation in severe phenotypes such as Hurler syndrome. In contrast, screening without a second-tier biochemical test may have a larger number of false-positives, along with increased detection of pseudodeficiency or late-onset variants. Together, these findings suggest that second-tier GAG testing preferentially enriches for clinically significant early-onset disease.

The enzyme assays lack specificity and, as such, fail to distinguish affected individuals from those with pseudodeficiencies [8]. Recent studies have shown that individuals homozygous for pseudodeficiency alleles in the *IDUA* gene are unlikely to be symptomatic and do not need any clinical follow up once pseudodeficiency status is confirmed [9]. It was believed that second-tier GAG testing can enhance screening resolution in cases where enzyme activity is low [10,11,12]. However, the data presented here suggest that internal disaccharide GAG analysis does not substantially improve the differentiation between pseudodeficiency cases and real MPS I cases where regular treatment and/or follow up is indicated. 

Interestingly, the proportion of attenuated cases remained nearly identical between cohorts (14% vs. 13%). This suggests that the use of second-tier GAG testing does not increase the risk of missing attenuated cases, despite previous work showing that GAG levels may remain low or only gradually increase in individuals with attenuated phenotypes [13]. Given that attenuated MPS I manifests as a clinically significant disease slower and later in childhood, it raises concern that some milder cases may not be reliably detected during the neonatal period with the current second-tier analysis.

One of the most significant differences between the two cohorts was the proportion of cases categorized with an unknown phenotype. Programs without second-tier GAG testing reported a substantially higher percentage of cases in this category (57%) compared to those with GAG testing (39%). This suggests that the inclusion of GAG testing may provide greater diagnostic clarity early in the evaluation process. Without substrate-based second-tier confirmation, states that use abnormal enzyme-based newborn screening with or without second-tier molecular testing may identify infants with pseudodeficiency or uncertain risk who do not have clinically meaningful substrate accumulation. Although definitive diagnosis and phenotype classification are established only after comprehensive confirmatory testing, incorporation of GAG analysis at the screening stage improves early risk stratification by identifying infants who require urgent evaluation. This refinement reduces the detection of undetermined cases where families are often left with ambiguous findings that complicate decisions around further testing and appropriate interventions. This limitation can lead to prolonged uncertainty and overmedicalization of children who may never even develop clinical symptoms. It will be important to continue to follow this cohort to identify if an early diagnosis changes the outcome of these possible attenuated cases.

A potential limitation of this study is the possibility of bias related to state-level differences in the prevalence of phenotypic outcomes, which may be influenced by genetic variation among populations and was not assessed as a part of this study.

## 5. Future Directions

Recent studies have emphasized the enhanced diagnostic ability of endogenous non-reducing end (NRE) GAG biomarkers over traditional internal disaccharide-based methods in NBS for MPS I [7,10,14]. Although internal disaccharide analysis has historically been the most widely used method within NBS programs, its reliance on enzyme digestion limits its specificity and can result in an overlap between affected and unaffected individuals. In comparison, the endogenous NRE method measures naturally occurring GAG fragments that accumulate at the enzyme blockage site in vivo, providing a clear biochemical marker of which enzyme is not functioning correctly [7]. Studies by Herbst et al. and Saville et al. demonstrated that NRE methods show a significantly greater differentiation between true-positive and false-positive cases [14,15]. Notably, no false-positives (other than pseudodeficiency) were shown across multiple MPS types. The data from our study and the previous studies from Herbst et al. denote that incorporating endogenous NRE GAG analysis instead of the currently used internal disaccharide method into second-tier testing algorithms may enhance the diagnostic precision of MPS I screening programs thereby reducing unnecessary follow-up while ensuring accurate early identification of both severe and attenuated cases.

## 6. Conclusions

Second-tier GAG testing improves the diagnostic accuracy of MPS I NBS by reducing false-positives and increasing the proportion of severe cases identified. Although the detection rates for attenuated cases were similar across cohorts, the greater proportion of undetermined outcomes in programs without GAG testing suggests that its benefit may be appreciated. Ongoing follow-up of these presumed attenuated cases will be essential in determining the full impact of incorporating GAG analysis into standard screening protocols.

However, given the illustrated superiority of endogenous dried blood spot-based GAG-NRE in comparison to the internal disaccharide GAG method used currently in differentiating severe from attenuated forms as well as pseudodeficiency cases, NBS programs should consider examining how the incorporation of GAG-NRE testing technology might improve diagnostic specificity [14]. As programs incorporate this technology, further work will be necessary to examine its performance in the public health domain.

## 7. Caveats

(1) Importantly, the designation of ‘false-positive’ and ‘false-negative’ test results in evaluating methodologies and algorithms for newborn screening programs is tremendously important to improve the efficiency and accuracy of this work. However, the relationship to clinical phenotype and the fate of the patient will not be known until decades have passed, and each patient’s clinical outcome is known. The current comparison intends to improve the screening methodology, yet we will not know the true ability of these methods to prognosticate medical outcomes for a few decades to come.

(2) Current newborn screening intends to find those infants with severe disease, for example patients with Hurler syndrome, who need expedient systemic therapy, i.e., hematopoietic stem cell transplant or gene therapy, to preserve the early trajectory of behavioral and cognitive potential, as well as minimize bone, heart, corneal, and other normal physiologic development. In the same process, infants predicted to have ‘attenuated’ disease still need treatment early in life. Those with Scheie syndrome develop disabling and painful skeletal and joint disease, corneal opacities with visual impairment, and life-limiting cardiac disease, and will be likely to have a shortened life span. Those with the intermediate Hurler-Scheie syndrome manifest even more severe and early problems such as severe short stature, painful and disabling kyphosis, joint contractures, cardiac and corneal disease and variable cognitive and psychiatric problems. Even these cases of attenuated disease suffer profound limitations in longevity and quality of life. Thus, all patients with MPS I, regardless of phenotype, deserve early treatment, with a screening methodology of sufficient sensitivity to minimize false-negative cases.

## Figures and Tables

**Table 1:** Overview of State-Based MPS I NBS Program Responses and Testing Methods.

Number of states/territories screening for MPS I as of August 2025	45
Number of responses received through Listserv	32
Number of states with incomplete responses/excluded in the study	7
Number of states that are included in the study	25
Number of states that have 1st tier IDUA enzyme assay	25
Number of states that have a 2nd tier biochemical assay (GAG/GAG + NGS)	8 (Three states were included in both categories: with data after implementation of GAG)
Number of states that have only molecular sequencing as a second-tier test	11
Number of states that do NOT have a second tier	9 (Three states were included in both categories: with data prior to implementation of GAG)
Number of states we did NOT get surveys back from	14

## Data Availability

The data supporting the findings of this study are available from the corresponding author upon request.

## References

[1] Clarke L.A., Adam M.P., Feldman J., Mirzaa G.M., Pagon R.A., Wallace S.E., Amemiya A. (1993). GeneReviews(^®^).

[2] Whitley C.B., Belani K.G., Chang P., Summers C.G., Blazar B.R., Tsai M.Y., Latchaw R.E., Ramsay N.K.C., Kersey J.H. (1993). Long-term outcome of Hurler syndrome following bone marrow transplantation. Am. J. Med. Genet..

[3] Orchard P.J., Gupta A.O., Eisengart J.B., Polgreen L.E., Pollard L.M., Braunlin E., Pasquali M., Lund T.C. (2022). Hematopoietic stem cell transplant for Hurler syndrome: Does using bone marrow or umbilical cord blood make a difference?. Blood Adv..

[4] Ojodu J., Singh S., Kellar-Guenther Y., Yusuf C., Jones E., Wood T., Baker M., Sontag M.K. (2018). NewSTEPs: The Establishment of a National Newborn Screening Technical Assistance Resource Center. Int. J. Neonatal Screen..

[5] Hall P.L., Sanchez R., Hagar A.F., Jerris S.C., Wittenauer A., Wilcox W.R. (2020). Two-Tiered Newborn Screening with Post-Analytical Tools for Pompe Disease and Mucopolysaccharidosis Type I Results in Performance Improvement and Future Direction. Int. J. Neonatal Screen..

[6] Aronovich E.L., Pan D., Whitley C.B. (1996). Molecular genetic defect underlying alpha-L-iduronidase pseudodeficiency. Am. J. Hum. Genet..

[7] Herbst Z.M., Urdaneta L., Klein T., Burton B.K., Basheeruddin K., Liao H.-C., Fuller M., Gelb M.H. (2022). Evaluation of Two Methods for Quantification of Glycosaminoglycan Biomarkers in Newborn Dried Blood Spots from Patients with Severe and Attenuated Mucopolysaccharidosis Type II. Int. J. Neonatal Screen..

[8] Bosfield K., Regier D.S., Viall S., Hicks R., Shur N., Grant C.L. (2021). Mucopolysaccharidosis type I newborn screening: Importance of second tier testing for ethnically diverse populations. Am. J. Med. Genet. A.

[9] Grady L.O., Zoltick E.S., Zouk H., He W., Perez E., Clarke L., Gold J., Strong A., Sahai I., Yeo J. (2025). Long-Term Health Outcomes of Individuals With Pseudodeficiency Alleles in IDUA May Inform Newborn Screening Practices for Mucopolysaccharidosis Type I. Am. J. Med. Genet. A.

[10] Herbst Z.M., Urdaneta L., Klein T., Fuller M., Gelb M.H. (2020). Evaluation of Multiple Methods for Quantification of Glycosaminoglycan Biomarkers in Newborn Dried Blood Spots from Patients with Severe and Attenuated Mucopolysaccharidosis-I. Int. J. Neonatal Screen..

[11] Tomatsu S., Montaño A.M., Oguma T., Dung V.C., Oikawa H., de Carvalho T.G., Gutiérrez M.L., Yamaguchi S., Suzuki Y., Fukushi M. (2010). Dermatan sulfate and heparan sulfate as a biomarker for mucopolysaccharidosis I. J. Inherit. Metab. Dis..

[12] Peck D.S., Lacey J.M., White A.L., Pino G., Studinski A.L., Fisher R., Ahmad A., Spencer L., Viall S., Shallow N. (2020). Incorporation of Second-Tier Biomarker Testing Improves the Specificity of Newborn Screening for Mucopolysaccharidosis Type I. Int. J. Neonatal Screen..

[13] Hendriksz C.J., Muenzer J., Vanderver A., Davis J.M., Burton B.K., Mendelsohn N.J., Wang N., Pan L., Pano A., Barbier A.J. (2015). Levels of glycosaminoglycans in the cerebrospinal fluid of healthy young adults, surrogate-normal children, and Hunter syndrome patients with and without cognitive impairment. Mol. Genet. Metab. Rep..

[14] Herbst Z.M., Kubaski F., Pollard L., Basheeruddin K., Burton B., Orsini J., Henderson M., Chakraborty P., Gelb M.H. (2025). High precision newborn screening for mucopolysaccharidosis type I by enzymatic activity followed by endogenous, non-reducing end glycosaminoglycan analysis. Mol. Genet. Metab..

[15] Saville J.T., Herbst Z.M., Gelb M.H., Fuller M. (2023). Endogenous, non-reducing end glycosaminoglycan biomarkers for the mucopolysaccharidoses: Accurate diagnosis and elimination of false positive newborn screening results. Mol. Genet. Metab..

